# Diagnostic Accuracy of Narrow-Band Imaging in Predicting Helicobacter pylori Gastritis in Patients With Dyspepsia

**DOI:** 10.7759/cureus.54756

**Published:** 2024-02-23

**Authors:** Hassan Liaquat Memon, Raja Taha Yaseen, Muhammad Ali Khalid, Ghulamullah Lail, Saleem Shahzad, Muhammad Manzoor Ul Haque, Ghazi Abrar, Shoaib Ahmed Khan, Syed Mudassir Laeeq, Nasir Hassan Luck

**Affiliations:** 1 Department of Hepatogastroenterology, Sindh Institute of Urology and Transplantation, Karachi, PAK; 2 Department of Medicine, Jinnah Medical & Dental College, Karachi, PAK

**Keywords:** narrow-band endoscopy, biopsy, endoscopy, helicobacter pylori, dyspepsia

## Abstract

Background

*Helicobacter pylori* (*H. pylori*) is one of the most prevalent causes of chronic gastritis that can lead to gastric cancer if left untreated. Currently, endoscopy and histology are the gold standard tests for the diagnosis of *H. pylori* gastritis. Recently, studies have shown the utility of narrow-band imaging (NBI) in predicting *H. pylori* gastritis. Therefore, we aimed to determine the diagnostic accuracy of NBI in predicting *H. pylori* gastritis in patients with dyspepsia.

Methodology

After obtaining approval from the Ethical Review Committee, Sindh Institute of Urology and Transplantation, this cross-sectional study was conducted in the outpatient Clinic of Hepatogastroenterology of the institute. Inclusion criteria involved all patients of either gender aged 18 to 65 years with dyspeptic symptoms. We excluded patients with a history of proton pump inhibitor use within two weeks before endoscopy, heart failure, previous gastrectomy, portal gastropathy, cirrhosis, use of antiplatelet medications, non-steroidal anti-inflammatory drugs or anticoagulant medication, and hemorrhagic or thrombophilia disorders. Each patient underwent endoscopy-guided NBI studies followed by biopsies from the antrum and body of the stomach. Multivariate logistic regression analysis was performed for the type of NBI pattern predicting *H. pylori* infection. The diagnostic accuracy was obtained individually for each NBI type and then for the presence of either two or all three NBI types in predicting *H. pylori* gastritis.

Results

Out of the total 775 patients enrolled in the study, abnormal NBI patterns were observed in 401 (51.7%) patients. The presence of abnormal NBI antral mucosal pattern on endoscopy was significantly associated with *H. pylori* infection (p < 0.001) with excellent diagnostic accuracy. Among the three NBI types, individually, NBI type III had excellent specificity and better diagnostic accuracy in predicting *H. pylori* gastritis than the other two types. Furthermore, the presence of all three abnormal NBI patterns (I+II+III) together was significantly associated with the presence of *H. pylori* gastritis with a sensitivity of 94.54%, specificity of 86.55%, and diagnostic accuracy of 90.32%.

Conclusions

NBI on endoscopy shows excellent diagnostic accuracy in identifying *H. pylori* gastritis in patients with dyspepsia. However, multicenter studies are required not only to validate our results but also to predict the pre-cancerous lesions on NBI in patients with *H. pylori* gastritis.

## Introduction

Dyspepsia is a commonly occurring condition that can be defined as the presence of one or more of the following symptoms: upper abdominal pain or discomfort, heartburn, regurgitation, dysphagia, and belching [1]. It is one of the most common presenting complaints in clinical practice, and *Helicobacter pylori* (*H. pylori*) is a well-known causative factor [2]. *H. pylori* is a spiral, micro-aerophilic, gram-negative, urease-producing rod affecting around 50% of patients worldwide presenting most commonly in the first decade [3]. In Pakistan, the prevalence rate of dyspepsia has been reported to be 65% compared to developed countries with a prevalence rate of around 40% [4,5]. It is an important etiological factor in the development of peptic ulcer disease, gastric adenocarcinoma, and gastric mucosa-associated lymphoid tissue lymphoma. The World Health Organization has also classified *H. pylori *as a Class 1 carcinogen [6]. Dyspeptic patients are more prone to malignancy if they have severe mucosal atrophy, intestinal metaplasia, and corpus-dominant active gastritis. Prompt diagnosis is not only essential for *H. pylori* eradication but also aids in relieving the dyspeptic symptoms and eliminating the risks of gastric malignancy [7].

Currently, multiple laboratory and histology-based tests are available for the detection of *H. pylori*, including *H. pylori* serology, stool for antigen detection, urea breath test, and invasive testing by endoscopy, histopathology, culture from tissue, and polymerase chain reaction. The sensitivity and specificity of invasive testing, i.e., endoscopic biopsy, for histology are 95% and 100%, respectively [8]. Recently, advancements in endoscopy and its techniques have led to the development of narrow-band imaging (NBI) which is an optical digital technique employed for the visualization of vessels and different patterns of gastric mucosa. It is used for differentiating benign from malignant tumors and aids in the risk stratification of invasive cancer [9]. Few Western studies have shown greater diagnostic accuracy of NBI with magnification in the detection of *H. pylori* with irregular patterns and reduced vessel density with a good sensitivity and specificity of 76% and 88%, respectively [7,10]. Three types of abnormal patterns related to *H. pylori* have been classified with a sensitivity and specificity of 95% and 48% for type I, 82% and 63% for type II, and 73.3 and 44% for type III, respectively [10,11]. The benefits associated with the use of NBI include reduced sampling error and early detection of malignant lesions [7]. Therefore, NBI can be cost-effective in situations where socioeconomic factors are an important determinant of a patient’s management [7].

As *H. pylori* infection is highly prevalent in our society and is an important factor causing significant morbidity, prompt diagnosis and eradication are essential to decrease the disease burden and eventually *H. pylori* gastritis [7].

No study has been performed in our part of the world to ascertain the relationship between the presence of *H. pylori* and endoscopic findings on NBI. Thus, in poor socioeconomic countries such as Pakistan, this study will help us decrease the disease burden of patients infected by *H. pylori* based on NBI and ultimately decrease the need for histology for the diagnosis of *H. pylori* gastritis. Therefore, we aimed to determine the diagnostic accuracy of NBI in predicting *H. pylori* gastritis in patients with dyspepsia.

## Materials and methods

After obtaining approval from the Ethical Review Committee (approval number: ERC-SIUT-167), this cross-sectional study was conducted in the outpatient Clinic of Hepatogastroenterology, Sindh Institute of Urology and Transplantation, Karachi from January 10, 2015, to December 31, 2018, after obtaining informed consent from patients. All patients aged 18 to 65 years of either gender with dyspeptic symptoms were included in the study. Patients with a history of proton pump inhibitor use within two weeks before endoscopy, heart failure (New York Heart Association grades III and IV), gastrectomy, portal gastropathy, liver cirrhosis, antiplatelet medications, non-steroidal anti-inflammatory drugs or anticoagulant medication, and hemorrhagic or thrombophilia disorders were excluded.

The sample size estimation was done by the institutional research statistician. The estimated sample size was 123 patients in six months keeping a 40% prevalence of *H. pylori* [4,5] and considering its sensitivity of 76% [7,11], specificity of 88% [7,11], and a desired precision of 10% along with 95% confidence interval. The sampling technique was non-probability consecutive sampling.

After obtaining informed consent, a detailed history and investigation were done to rule out exclusion criteria. All cases were subjected to clinical criteria and endoscopic evaluation. Upper gastrointestinal endoscopy was performed after an overnight fast of eight hours. Esophagogastroduodenoscopy (EGD) was done by the researcher under the supervision of an expert endoscopist with experience of more than 1,000 NBI cases by a video-endoscope (GIF-H 190 Olympus). The light source in NBI was opened and examined for different abnormal patterns and vasculature of gastric mucosa. After identifying different mucosa patterns, endoscopic photographs were obtained followed by biopsies from the antrum and body of the stomach. Biopsy specimens were immersed in formalin and sent for histopathological examination by an expert histopathologist with more than 20 years of experience in gastrointestinal and infectious diseases. Data were collected and dyspepsia was labeled as the presence of any one of the following symptoms for at least one week, assessed clinically based on the medical history of the patient: upper abdominal pain/discomfort, heartburn, regurgitation, dysphagia, and belching. Data collected was transferred to a pre-designed proforma by the researcher. As per institutional policy, all tests were performed free of cost.

Statistical analysis was done by SPSS version 24 (IBM Corp., Armonk, NY, USA). Data were presented as mean and standard deviation for continuous variables such as age in years and duration of disease. While the frequencies and percentages were calculated for categorical variables such as gender, *H. pylori*, and NBI. The continuous variables were analyzed using the Student’s t-test, while categorical variables were analyzed using the chi-square test. A p-value <0.05 was considered statistically significant. Multivariate logistic regression analysis was performed for the type of NBI pattern predicting *H. pylori* infection. Sensitivity, specificity, positive predictive value (PPV), negative predictive value (NPV), and diagnostic accuracy were obtained individually for each NBI type and then for the presence of either two or all three NBI types in predicting *H. pylori* gastritis.

## Results

Of the total 775 patients enrolled in the study, most were males (474, 61.2%). The mean age was 38.4 ± 12.6 years, with most aged <40 years (Table 1). Comorbidities were observed in 113 (14.6%) patients, with diabetes mellitus and hypertension noted in 52 (6.7%) and 34 (4.4%) patients, respectively.

**Table 1:** Baseline characteristics of the population included in the study (n = 775). NSAIDs = non-steroidal anti-inflammatory drugs

Study population		n (%)
Mean age (years ± S.D)		38.4 ± 12.6
Age less than 40 years		486 (62.7)
Gender	Males	474 (61.2)
	Females	301 (38.8)
Comorbidities	Diabetes	52 (6.7)
	Hypertension	34 (4.4)
	Others	27 (3.5)
	No comorbidities	662 (85.4)
History of NSAIDs usage	Yes	85 (11)
	No	690 (89)
Disease duration	<1 month	101 (13)
	>1 month	674 (87)
Symptoms of dyspepsia at baseline	Epigastric pain	601 (77.5)
	Bloating	119 (15.4)
	Belching	71 (9.2)
	Early satiety	132 (17)
	Heartburn	73 (9.4)
Narrow-band imaging	Normal	374 (48.3)
	Abnormal	401 (51.7)
*Helicobacter pylori* on histology	Present	366 (47.2)
	Absent	409 (52.8)
Narrow-band imaging pattern	Type I	112 (14.5)
	Type II	129 (16.6)
	Type III	160 (20.6)

The duration of dyspepsia symptoms for more than one month was observed in 674 (87%) patients. The most common presenting symptoms were epigastric pain in 601 (77.5 %) patients, followed by early satiety in 132 (17%), bloating in 119 (15.4%), heartburn in 73 (9.4%), and belching in 71 (9.2%). The most common endoscopy finding was gastric erythema noted in 482 (62.2%) patients. Gastric erosions were observed in 112 (14.4%) patients, while endoscopy was normal in the remaining patients. *H. pylori* infection was noted on gastric biopsy in 366 (47.2%) patients presenting with dyspepsia, of whom most were males (224, 61.2%).

Different patterns of gastric mucosa were identified on NBI. Abnormal NBI patterns were noted in 401 (51.7%) patients. Of those with abnormal NBI patterns, the most common was type III was observed in 160 (39.9%) patients, while type II and type I patterns were noted in 129 (32.1%) and 112 (27.9%) patients, respectively. The normal gastric mucosal pattern on NBI, i.e., type 0, was observed in 374 (48.3%) patients (Table 1). On histopathological evaluation, *H. pylori* was identified in 366 (47.2%) biopsies, of which 346 (94.5%) patients had abnormal NBI findings on endoscopy (Table 2).

**Table 2:** Comparison of NBI and its types in predicting Helicobacter pylori gastritis (n = 775). NBI = narrow-band imaging

Variable		*Helicobacter pylori* on gastric biopsy		P-value
		Present (n = 366), n (%)	Absent (n = 409), n (%)	
NBI	Abnormal	346 (94.5)	55 (13.4)	≤0.001
	Normal	20 (5.5)	354 (86.6)	
NBI type I	Abnormal	101 (27.6)	11 (2.7)	≤0.001
	Normal	265 (72.4)	398 (97.3)	
NBI type II	Abnormal	109 (29.8)	20 (5)	≤0.001
	Normal	257 (70.2)	389 (95)	
NBI type III	Abnormal	136 (37.2)	24 (5.9)	≤0.001
	Normal	230 (62.8)	385 (94.1)	

The presence of abnormal NBI antral mucosal pattern on endoscopy was significantly associated with *H. pylori* infection (p < 0.001), with a sensitivity, specificity, PPV, NPV, and diagnostic accuracy of 94.54%, 86.5%, 86.3%, 94.7%, and 90.32%, respectively (Table 3).

**Table 3:** Diagnostic accuracy of NBI in predicting Helicobacter pylori gastritis. PPV = positive predictive value; NPV = negative predictive value; NBI = narrow-band imaging

Statistics	Value	95% confidence interval
Sensitivity	94.54%	91.69% to 96.63%
Specificity	86.55%	82.86% to 89.71%
PPV	86.3%	82.52% to 89.50%
NPV	94.65%	91.86% to 96.7%
Diagnostic accuracy	90.32%	88.02% to 92.31%

On the multivariate logistic regression analysis, all NBI patterns were significant predictors of *H. pylori* gastritis with the highest odds ratio (OR) for type I NBI pattern (OR = 162.5, p < 0.001), followed by type III (OR = 100.3, p < 0.001) and type II NBI patterns (OR = 96.4, p < 0.001) (Table 4).

**Table 4:** Multivariate Cox regression analysis of NBI type predicting Helicobacter pylori gastritis. NBI = narrow-band imaging

Variables	P-value	Hazard ratio	95% confidence interval	
			Lower limit	Upper limit
NBI type I	≤0.001	162.5	75.4	350.4
NBI type II	≤0.001	96.45	50.1	185.9
NBI type III	≤0.001	100.3	53.7	187.5

Among the three NBI types, NBI type III had excellent specificity and better diagnostic accuracy in predicting *H. pylori* gastritis than the other two types. All three NBI types lacked sensitivity on an individual basis. However, the presence of NBI types I and III together had a better specificity and diagnostic accuracy for *H. pylori* gastritis, with a sensitivity of around 73%. Furthermore, the presence of all three abnormal NBI patterns (I+II+III) together was significantly associated with the presence of *H. pylori* gastritis, with a sensitivity of 94.54%, specificity of 86.55%, and diagnostic accuracy of 90.32% (Table 5).

**Table 5:** Diagnostic accuracy of types of NBI in predicting Helicobacter pylori gastritis. PPV = positive predictive value; NPV = negative predictive value; NBI = narrow-band imaging

Statistics	NBI type I	NBI type II	NBI type III	NBI type I+II	NBI type II+III	NBI Type I+III	NBI type I+II+III
Sensitivity	27.6%	29.78%	37.16%	57.38%	66.94%	64.75%	94.54%
Specificity	97.3%	95.11%	94.13%	92.42%	89.24%	91.44%	86.55%
PPV	90.18%	84.5%	85%	87.14%	84.78%	87.13	86.3%
NPV	60.03%	60.22%	62.6%	70.79%	75.10%	74.35%	94.65%
Diagnostic accuracy	64.38%	64.26%	67.23%	75.87%	78.71%	78.84%	90.32%

## Discussion

Worldwide, *H. pylori* infection has a prevalence of around 50% with increasing prevalence in developing countries, especially among populations with low socioeconomic status, low educational level, and living in crowded environments [3,12]. Furthermore, the prevalence of infection is higher in the dyspeptic population compared to their asymptomatic counterparts [13-14]. In our study, the endoscopic frequency of *H. pylori* infection among the symptomatic population was 47% with slight male predominance. Low socioeconomic status, low level of education, and living in congested crowded environments may explain this high frequency of infection among the dyspeptic population in a developing country like Pakistan. It is usually acquired in childhood and its prevalence increases with age. Shi et al. [15] reported the peak prevalence of *H. pylori* infection (67%) in the fourth decade of life among the Chinese population. Our study has also demonstrated the highest frequency of infection in patients below the age of 40 years.

Yamamichi et al. [16 ] used endoscopy and double-contrast upper gastrointestinal barium studies for the detection of atrophic gastritis and reported the association between four grades of atrophic gastritis and *H. pylori* infection. Similarly, Cakmakci et al. [17] reported the correlation between *H. pylori* and endoscopic and sonographic clues for the diagnosis of antral gastritis. In contrast, we reported, for the first time in Pakistan, an association between *H. pylori* infection and NBI.

NBI is an imaging technique with the enhancement of the optical image of different abnormal patterns and vessels of gastric mucosa with a better view reflecting gastritis and other benign and malignant conditions. Abnormal patterns of gastric mucosa associated with *H. pylori* infection were divided into three types [9-11]. Previously, Cho et al. [18] demonstrated an excellent diagnostic accuracy of 93.3% of NBI in detecting *H. pylori* gastritis in the Chinese population. Similarly, Glover et al. revealed similar results in the European population [19].

The results from our study also revealed similar results to earlier studies with excellent sensitivity, specificity, and diagnostic accuracy of NBI in the detection of *H. pylori* gastritis (Table 3). However, contrary to the study by Tahara et al. [10] in the Japanese population, the NBI types I, II, and III had excellent specificity individually in predicting *H. pylori* gastritis but lacked sensitivity. However, the sensitivity increased by the combination of NBI types with the highest sensitivity for all three NBI types (I+II+II) followed by NBI type I+III (Table 5).

These differences in results can be attributed to the different demographics of our population compared to those discussed in previous studies. One of the major strengths that can be attributed to our study is the large sample size. Second, it was a pioneering study from Pakistan describing the utility of NBI in predicting *H. pylori* gastritis. There were certain limitations of our study. First, it was a single-center study and we only enrolled patients presenting with dyspepsia in our outpatient department. Second, interobserver variability between endoscopists in describing different patterns of NBI on endoscopy cannot be ruled out. Third, the resolution of NBI findings on endoscopy after *H. pylori* eradication therapy was not observed. Fourth, due to the unavailability of the rapid urease test at our center, it was not performed on the biopsied samples.

## Conclusions

NBI on endoscopy has shown excellent diagnostic accuracy in identifying *H. pylori* gastritis in patients with dyspepsia. However, multicenter studies are required not only to validate our results but also to predict the association of pre-cancerous and cancerous lesions on NBI in patients with *H. pylori* gastritis.
